# Evaluation of Microscopic Tumour Extension in Localized Stage Non-Small-Cell Lung Cancer for Stereotactic Radiotherapy Planning

**DOI:** 10.3390/cancers14051282

**Published:** 2022-03-02

**Authors:** Martin Schmitt, Lucie Aussenac, Joseph Seitlinger, Véronique Lindner, Georges Noël, Delphine Antoni

**Affiliations:** 1 Radiotherapy Department, Strasbourg Europe Cancer Institute, 17 Rue Albert Calmette, 67200 Strasbourg, France; ma.schmitt@icans.eu (M.S.); d.antoni@icans.eu (D.A.); 2 Pathology Department, Hautepierre University Hospital, 1 Rue Molière, 67000 Strasbourg, France; lucie.aussenac@chru-strasbourg.fr (L.A.); veronique.lindner@chru-strasbourg.fr (V.L.); 3 Thoracic Surgery Department, Lorraine Heart and Vessel Institute, 5 Rue du Morvan, 54500 Vandœuvre-lès-Nancy, France; joseph.seitlinger@chru-strasbourg.fr

**Keywords:** non-small-cell lung carcinoma, adenocarcinoma, squamous cell carcinoma, stereotactic body radiation therapy

## Abstract

**Simple Summary:**

Stereotactic radiotherapy for localised stage non-small-cell lung carcinoma is an alternative indication for patients who are inoperable or refuse surgery. A study showed that the microscopic tumour extension of non-small-cell lung carcinoma varied according to the histological type, which allowed us to deduce adapted margins for the clinical target volume. The objectives of our retrospective study are. The objective of our study is to measure the microscopic tumour extension of T1N0 or T2aN0 primary lung tumors who underwent surgery. The margin required to cover the microscopic tumour extension with a 95% probability is 4.4 mm and 2.9 mm for squamous cell carcinoma and adenocarcinoma, respectively. Multivariate analysis showed a statistically significant relationship between the maximum microextension distance and size with the shrinkage coefficient.

**Abstract:**

Background: Stereotactic radiotherapy for localised stage non-small-cell lung carcinoma (NSCLC) is an alternative indication for patients who are inoperable or refuse surgery. A study showed that the microscopic tumour extension (ME) of NSCLC varied according to the histological type, which allowed us to deduce adapted margins for the clinical target volume (CTV). However, to date, no study has been able to define the most relevant margins for patients with stage 1 tumours. Methods: We performed a retrospective analysis including patients with adenocarcinoma (ADC) or squamous cell carcinoma (SCC) of localised stage T1N0 or T2aN0 who underwent surgery. The ME was measured from this boundary. The profile of the type of tumour spread was also evaluated. Results: The margin required to cover the ME of a localised NSCLC with a 95% probability is 4.4 mm and 2.9 mm for SCC and ADC, respectively. A significant difference in the maximum distance of the ME between the tumour-infiltrating lymphocytes (TILs), 0–10% and 50–90% (*p* < 0.05), was noted for SCC. There was a significant difference in the maximum ME distance based on whether the patient had chronic obstructive pulmonary disease (COPD) (*p* = 0.011) for ADC. Multivariate analysis showed a statistically significant relationship between the maximum microextension distance and size with the shrinkage coefficient. Conclusion: This study definitively demonstrated that the ME depends on the pathology subtype of NSCLC. According to International Commission on Radiation Units and Measurements (ICRU) reports, 50, 62 and 83 CTV margins, proposed by these results, should be added to the GTV (Gross tumour volume). When stereotactic body radiation therapy is used, this approach should be considered in conjunction with the dataset and other margins to be applied.

## 1. Introduction

Non-small-cell lung carcinomas (NSCLCs) represent 85% of lung cancer diagnoses [1]. Only 15% to 25% of tumours are diagnosed early [2,3], and their management is considerably improved with the development of stereotactic body radiation therapy (SBRT), mini-invasive surgery and interventional radiology.

The standard treatment for stage I NSCLC is surgery, reaching 3-year and 5-year overall survival rates of 77.9–79% and 66.1–84%, respectively [4,5,6]. However, approximately one in four patients is not eligible for surgery due to medical contraindications or because the patient refuses the operation.

Currently, the approved therapeutic alternative is SBRT, allowing a 3-year local control rate of 89–96%, a specific survival rate of 66–82% and an overall survival rate of 32–91.8% [7,8,9,10,11].

To delineate targets in radiotherapy, several volumes have been defined by the International Commission on Radiation Units and Measurements (ICRU) reports 50, 62 and 83, including the gross tumour volume (GTV), the clinical target volume (CTV) including microscopic extension (ME) and the planning target volume (PTV). CTV remains a disputable topic in SBRT studies.

A study of CTV margins for lung cancer treated at the time of tridimensional radiotherapy was performed based on pathological examination data [12]. However, to date, no studies have defined specific margins for tumours meeting the criteria for SBRT. These margins remain to be specified to optimise the definition of irradiated volumes, to improve local control and to limit side effects [13,14]. The objective of our study was therefore to use anatomopathological slices to evaluate bronchopulmonary tumour cell extension beyond the macroscopically visible tumour to determine the best CTV margins for SBRT.

## 2. Materials and Methods

### 2.1. Ethical Approval

This study follows the mandatory French laws required by the CNIL (Commission Nationale de l’informatique et des libertés), was declared to this French institution by the MR004 form and was recorded in the HDH (Health Data Hub) and was approved by our Institutional Review Board.

### 2.2. Patients

One hundred and twelve patients with localised pT1N0 or pT2aN0 adenocarcinoma (ADC) and 42 patients with localised pT1N0 or pT2aN0 squamous cell carcinoma (SCC), who underwent surgery from January 2014 to December 2018 and from January 2013 to December 2018, respectively, were retrospectively included. Patients with neoadjuvant chemotherapy or without a preoperative computed tomography (CT) scan with iodinated contrast injection were excluded. A keyword search in the computerised database of anatomoclinical reports (DIAMIC) was performed to identify cases that matched the following inclusion criteria over the chosen period: “(p) T1 (a b c) T2a” AND “adenocarcinoma lung” OR “squamous cell carcinoma” OR “non small cell lung cancer”. For each group of pathologies, the patients’ characteristics are reported in Table 1.

Surgery consisted of a segmentectomy, lobectomy or pneumonectomy by thoracotomy or videothoracoscopy with extemporaneous examination and lymph node dissection. Surgical specimens were sent fresh and oriented to the pathology department.

The superoinferior, anteroposterior, medial–lateral, and maximal dimensions of each tumour were measured on the preoperative CT scan by a radiologist and a radiation oncologist using optimal window/level settings and then compared with the tumour size measured on pathology.

### 2.3. Histology

**Figure 1:**
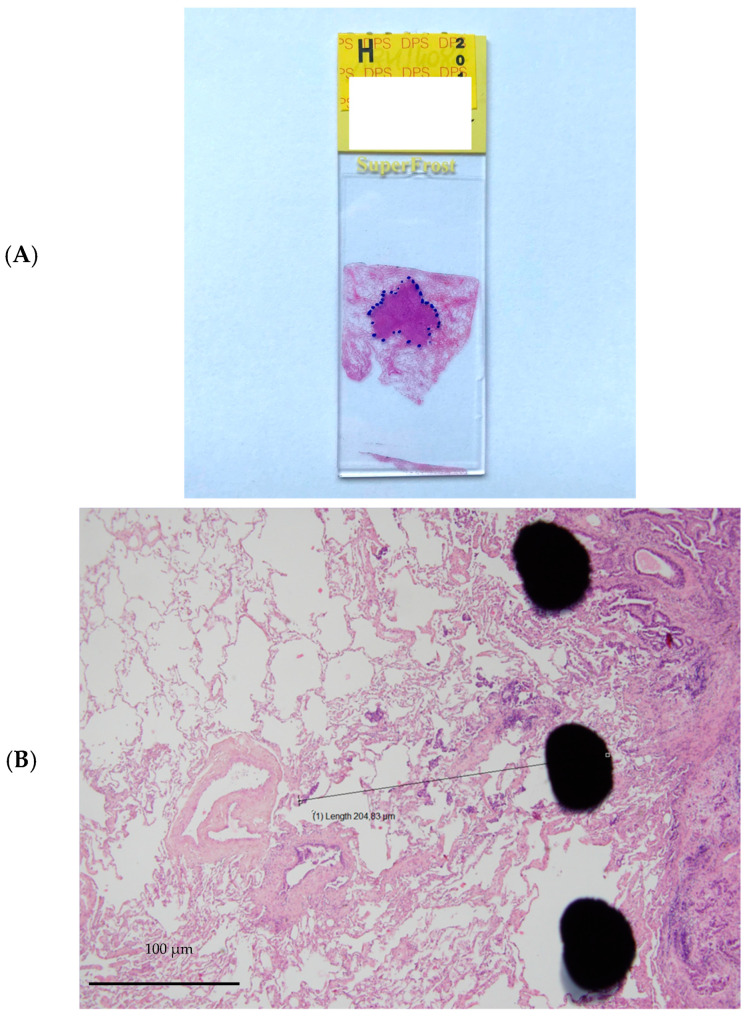
Example of microinvasion measurement. (**A**). Tumour stained with hematoxylin and eosin and delineated with marker pen. (**B**). Microscopic tumour extension measured from the boundary between the tumour and healthy tissue to the most distant tumour cell using a micrometre.

The resected specimens were fixed in 10% buffered formalin for 24 h, dehydrated in successive alcohol baths and then embedded in paraffin. The paraffin blocks were cut with a microtome, and the bands were spread on slides and then stained with haematoxylin-eosin. The thickness of the slices was 4 microns. All slices involving the tumour were analysed, representing one to 6 slices per patient with an average of 3 slices. The tumour was delineated with the naked eye using a marker (Figure 1). Then, the microscopic tumour extension (ME) was measured on the slices from the boundary between the tumour and healthy tissue to the most distant tumour cell using a micrometre. For each patient, only the “longest” distance from the available and studied slices was retained for the final analysis. The measurement performed was weighted by a factor corresponding to the shrinkage coefficient of the tissue related to the different fixation and inclusion steps. In a prospective monocentric study, Park et al. described that formalin fixation caused 4.06% shrinkage in 46.8% of tumours. The overall mean tumour size change after formalin fixation was 0.77 mm (SD: 1.02 mm), and the percentage difference in tumour size was 4.06% (range: 0–26.1%; SD: 5.15%) [15]. Measured size, type of architecture according to the World Health Organization (WHO) 2015 classification [16], presence or absence of angioinvasion, lymphatic invasion, presence of fibrosis, inflammation or haemorrhage, and tumour-infiltrating lymphocytes (TILs) were also analysed. TIL rates were used to define three groups: 0–10%, 20–40%, and 50–90% [17].

### 2.4. Statistical Analysis

Statistical analysis was performed using R v3.6.0 software. The Student’s *t* test was used to analyse the relationship between the ME and the different clinical or histological parameters. The Chi2 or Fisher exact test was used to compare qualitative parameters. The analysis of the margins required to recover the ME was performed by comparing the percentage of patients with an ME less than or equal to the value of the analysed margin. In the case of a recovery of 95% of the sample, the margin is considered acceptable. Concerning the multivariate analyses, the distribution of the residuals did not follow a normal distribution, and we calculated confidence intervals and *p* values using the bootstrap method (1000 iterations).

## 3. Results

### 3.1. Patient and Tumour Characteristics

The patient and tumour characteristics are presented in Table 1. The mean age at diagnosis of the ADC and SCC groups was 65.5 years (SD 8.29) and 65.8 years (SD 7.84), respectively. In the ADC and SCC groups, the F/M ratios were 1.15 and 0.27, respectively. In the ADC and SCC groups, 34 patients (30%) and 19 patients (45%) developed chronic obstructive pulmonary disease (COPD), respectively.

The mean sizes of ADC and SCC tumours on the CT scan were 2.15 cm (SD 0.9; min = 0.8; max = 5.3) and 2.33 cm (SD 1.1; min = 0.3; max = 5.4), respectively (Table 2).

Three hundred forty-one slices of ADC were derived from 112 tumours, and 127 slices of SCC were derived from 42 tumours. The mean sizes of ADC and SCC tumours were 1.97 cm (SD 0.85; min = 0.21; max = 3.95) and 2.46 cm (SD 1.00; min = 1.04; max = 4.16), respectively (Table 2).

### 3.2. Radio-Histologic Correlations

**Figure 2:**
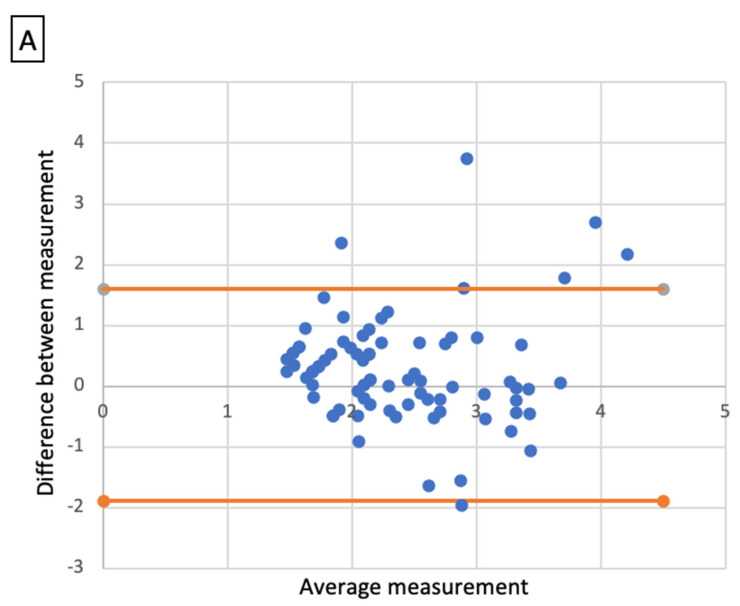

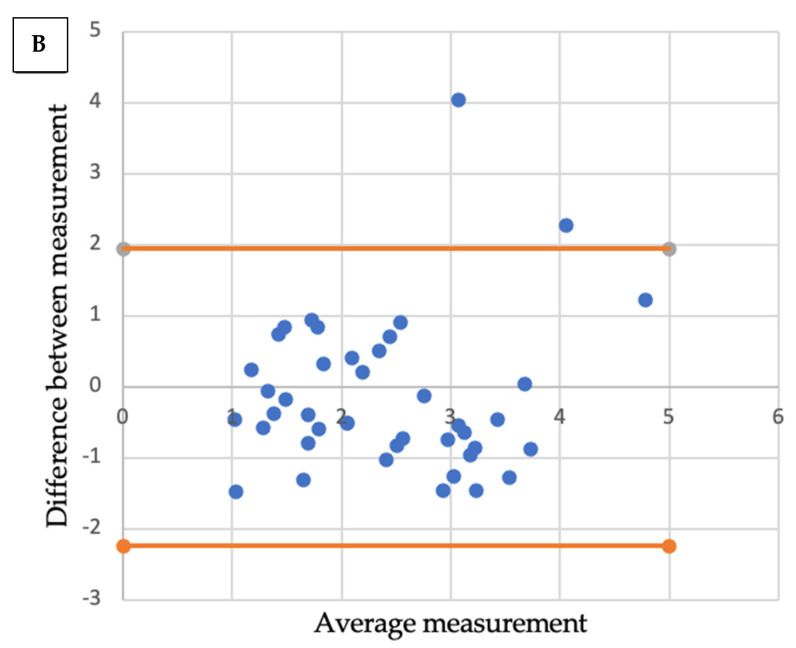
Bland-Altmann plots. Radiologic size and histologic size with shrinkage coefficients for adenocarcinoma (**A**) and squamous cell carcinoma (**B**).

Comparative analysis showed a significant correlation between radiologic size and histologic size with shrinkage coefficients for ADC and SCC, respectively (*p* < 0.001 and *p* < 0.01). A Bland–Altmann plot was made respectively for each histological type between radiologic size and histologic size with shrinkage coefficients (Figure 2).

### 3.3. Adenocarcinoma

**Figure 3:**
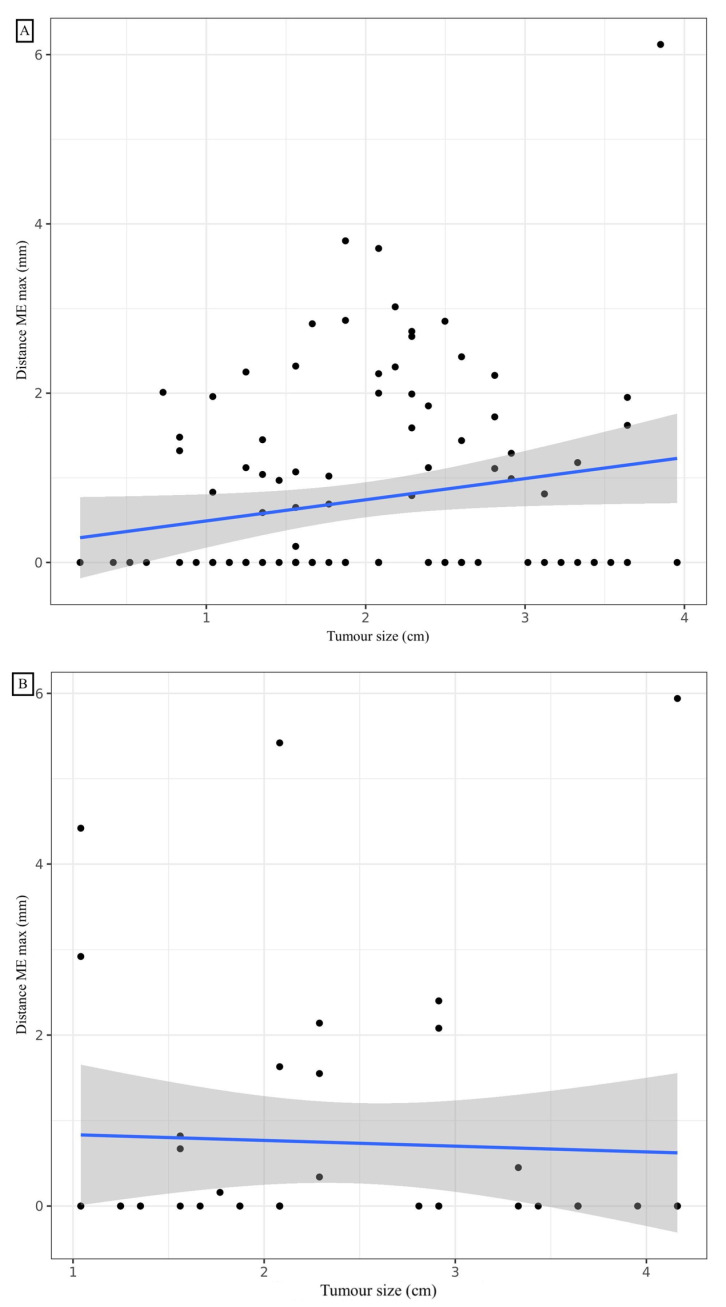
Correlation between maximal distance microextension and tumour size for adenocarcinoma (**A**) and squamous cell carcinoma (**B**).

A mean distance of 0.73 mm (standard deviation: 1.12), a median of 0 mm (0; 1.30), and minimum and maximum values of 0 mm and 6.12 mm, respectively, were observed for the ME (Table 2). Considering a margin of 3 mm, the ME coverage rate was 96.4%, which was not significantly different from 95% (*p* = 0.488). To cover exactly 95% of the sample, a margin of 2.86 mm would be required. The affected lobe, the presence of TILs, and the presence of vascular or bronchial contact did not significantly influence the ME. A statistically significant linear correlation was noted between the maximum ME distance and the number of slides (correlation coefficient: 0.233, 95% CI (0.0493; 0.401); *p* = 0.014) (Figure 3). A significant difference in distance from the ME depending on whether the patient had COPD (*p* = 0.011). The mean rank of ME was not significantly different based on the GOLD stage (*p* = 0.062). To cover 95% of the ADC sample with COPD, a margin of 3.74 mm would be required. To cover 95% of the ADC sample without COPD, a margin of 2.27 mm would be required. At a risk of 5%, when adjusting for COPD, the number of slides analysed, TILs and GTV, we were unable to show a statistically significant relationship between the maximal distance ME and size with the shrinkage coefficient (Table 3).

### 3.4. Squamous Cell Carcinoma

A mean distance of 0.74 mm (standard deviation: 1.49), a median of 0 mm (0; 0.62), and minimum and maximum values of 0 mm and 5.94 mm, respectively, were observed for the ME (Table 2). Considering a margin of 3 mm, the ME recovery rate was 92.9%, which was not significantly different from 95% (*p* = 0.524). To cover exactly 95% of the sample, a margin of 4.43 mm is required. COPD and the affected lobe, and the presence of vascular or bronchial contact did not significantly influence the presence and distance of the ME. The mode of dissemination, including vascular, lymphatic or endobronchial dissemination, did not influence the distance of the ME. The presence of TILs was significantly inversely correlated with the distance from the ME. Post hoc analysis of the comparison of the three TIL groups showed a significant difference in maximum ME distance between the 0–10% and 50–90% (*p* < 0.05) TIL groups. To cover 95% of the samples in the TIL 0–10%, 20–40% and 50–90% groups, margins of 4.97 mm, 2.13 mm and 0 mm, respectively, would be required. With a 5% risk, by adjusting for TILs, COPD, the number of slides analysed and GTV volume, a statistically significant relationship was noted between the maximal distance ME and size with the shrinkage coefficient. Distance ME was also significantly linked to TILs and GTV volume for SCC (Table 4). To cover exactly 95% of SCCs greater than 1.5 cm long on the CT, a margin of 4.92 mm is required. To cover exactly 95% of SCC less than or equal to 1.5 cm long axis on CT, a margin of 0.74 mm is required.

## 4. Discussion

There are limited data to directly determine whether the radiologic tumour size, as defined by computed tomographic (CT) imaging, correctly represents the gross pathological tumour size in NSCLC. Giraud et al. reported that without ME, the radiologic size of a lung tumour was very close to its gross pathological size [12]. Similarly, Li et al. demonstrated that the three-dimensional measurement of GTV on CT approximated its pathologic size, not including ME [18]. In contrast, Chan et al. suggested that the radiographic tumour size overestimates the pathologic size [19]. The correlation between radiologic size and pathologic size validates the marked pen limit that we delineated and beyond which we measured ME.

To our knowledge, we present the largest study in terms of patient number and in terms of analysed relevant slices to evaluate tumour cell diffusion beyond the carcinoma boundaries. Few data have been published on the ME or the most appropriate CTV margins during lung irradiation. Giraud et al. showed that margins of 8 mm and 6 mm around the GTV for ADC and SCC, respectively, covered 95% of the ME. This study included 70 patients with ADC (32 patients) or SCC (38 patients), stage I to IV. A mean of 5 slices were analysed per patient (354 slices in total). Notably, in the study of Giraud et al., only 46% of patients had a stage I tumour. Consequently, the majority of tumours were not indicated for SBRT. Furthermore, if the ME is related to tumour size, the study by Giraud et al., with larger tumours, may overestimate the ME of stage I tumours [12]. Li et al. showed that for a 95% coverage of the ME, the margins to be applied for CTV were 7 mm and 5 mm for ADC and SCC, respectively. The content of the article was only accessible to Chinese-reading people, limiting data analysis; this study included a total of only 43 patients [18]. The study by Yuan et al. was conducted to quantify the extent of ME beyond nodal GTV. The extent of nodal extracapsular extension (ECE) on pathologically dissected lymph nodes of 243 patients with NSCLC was measured, and the correlation between ECE and lymph node sizes, histological type and tumour cell differentiation was studied. A 3 mm- and 8 mm-GTV margin was proposed for lymph node sizes ≤ 20 mm and ≥20 mm, respectively [20]. Van Loon et al. demonstrated a correlation between ME and the volume of GTV delineated on the scanner in a study including 34 NSCLC patients. Histological types were variable: 18 ADCs, six SCCs, four large cell carcinomas, three mixed adeno-squamous carcinomas, one bronchioloalveolar tumour and two NSCLC not otherwise specified. Furthermore, this study included NSCLCs with a large range of diameters from 11.1 to 84.8 mm [21]. Grills et al. showed that tumour grade was correlated with ME in 35 patients with ADC. However, this study did not consider tissue shrinkage due to fixation and included T1N0 tumours. However, looking at the range of tumour sizes, we noticed that the minimum and maximum sizes were 8 mm and 48 mm, respectively, which corresponds to T1 to T2 tumours [22]. As our study included only stage I NSCLC, these correlations between tumour size and ME could explain the difference observed between our results and those reported by Giraud et al., and Li et al. [12,18]. The hypothesis that can explain the lower ME measured in the current study is the relationship between tumour size and the size of the ME. Because the range of size of the tumour included in this current study was not so large and due to limited sample size, we failed to demonstrate a significant correlation between tumour size and ME for adenocarcinoma. We found a significant association between histologic tumour size and ME for SCC. Having also shown a significant correlation between radiographic and histologic size, we evaluated the margin required to cover 95% of MEs when the longest tumour axis measured on CT is greater than or less than 1.5 cm.

Significant heterogeneity in the definition of volumes and dose prescription for stereotactic pulmonary radiotherapy is noted. The ICRU 91 has not specified a value for the margin defining CTV; however, it recommends defining a CTV in each case. The European Society of Radiation Oncology (ESTRO) 2017 recommendations showed that of the 11 institutions surveyed, all but one did not apply a margin for CTV and applied a median margin of 5 mm (3–7 mm) for PTV [23]. Senthi et al. also highlighted a significant heterogeneity of target volumes in their literature review of 20 studies. PTV was defined as a 3- to 11-mm margin from internal target volume (ITV). They were sometimes split into 3- to 8-mm margins from GTV to CTV and 3- to 5-mm margins from CTV to PTV [24]. Recently, in a retrospective study, Trémolières et al. showed that personalised PTV margins of 4 mm can be applied for upper lobe lesions located above the carina if only a single 4DCT is performed [25].

The systematic review by Chi et al. highlighted significant heterogeneity in the prescription of the total dose, fractionation and isodose. A biological equivalent dose (BED) at the isocentre of at least 100 Gy_10_ was associated with better survival and local control. However, the authors also showed that the peripheral BED ranged from 37.5 to 211.2 Gy_10_ and was less than 100 Gy_10_ in many studies [26]. In contrast, Wulf et al. found that the peripheral BED of the target volume was an independent factor influencing local control [27]. Klement et al. showed that the average between near-minimum and near-maximum doses (BEDave) was better correlated with tumour control probability than either BEDmax or BEDmin, and was highly correlated with the mean GTV dose. The authors concluded that BEDave may be used as a prescription target and proposed and suggested that more attention should be given to high mean doses within the GTV than to the coverage of the PTV [28]. These differences in margins and dose prescription may lead to variations in ME coverage. Of note, the use of a margin for ITV or PTV could cover these microextensions; however, it is important to remember that the goal of the PTV is to consider the different errors and uncertainties and not the ME, which is the role of the CTV or the differences in motion, which is the role of ITV. Finally, the application of a heterogeneous dose prescription with its dose fall-off at the CTV/PTV border is also a relevant point to discuss. Grills et al. showed that the variability of ME coverage can be substantially dependent on the planned dose distribution [22]. Finally, even if the CTV is generally undefined, assuming coverage of the ME by the penumbra, ICRU 91 recommends that the CTV be formally defined. Moreover, ICRU 91 reminds us that the penumbra can be asymmetrical and that two treatment plans delivering the same dose to the GTV can deliver two different doses to the CTV [29].

The current results demonstrate an inverse correlation between TILs and ME in the SCC group. The presence of TILs is a prognostic factor for prolonged survival in several cancers, including NSCLC [30,31,32,33]. The literature review by Bremnes et al. revealed a recent interest in TILs. Of 17 studies on the impact of TILs in NSCLCs, 13 were published after 2011. Donnem et al. showed that CD8+ T cell density was a positive and independent prognostic factor for relapse-free, specific and overall survival [34]. The meta-analysis by Geng et al. analysing 29 articles for a total of 8600 patients showed that CD8+ T cell infiltration of the tumour stroma and microenvironment was associated with improved overall survival. CD4+ T cell infiltration of the tumour stroma was also associated with improved overall survival [35]. Our results suggest that ME is blocked by TILs; consequently, the CTV should be adjusted according to the TIL rate. TIL biopsy research could help to adjust CTV margins. Few studies have compared TIL rates on biopsy and resection specimens. In the breast, two studies have shown that the TIL rate evaluated on biopsy may be representative of the whole tumour [36,37]. However, discordant results were found in colorectal cancer [38]. To our knowledge, no such comparison has been made in NSCLC. Furthermore, biopsy raises a new issue. Aspiration specimens are contaminated by blood, and the degree of contamination varies. The number of lymphocytes in the smear does not always reflect the grade of infiltration of TILs. Nakahara et al. showed that the ratio of the number of lymphocytes to the number of lymphocytes and neutrophils was correlated with the TIL rate [39], so this could be a solution for patients with only a biopsy. However, the biopsy is not systematic before NSCLC SBRT, and a radiomics approach should be considered. Many studies have demonstrated the feasibility of radiomic prediction of the immune microenvironment using CT imaging in NSCLC [40,41,42]. These methods could make it possible to adapt CTV by describing the tumour microenvironment without invasive methods. However, before the validation of radiomics in microenvironmental analysis by large trials, we propose to use the greater margins when the TIL rate is not known.

The impact of COPD status on ME in NSCLC patients has already been demonstrated in other studies. Maeda et al. showed less differentiated, more invasive histological profiles for stage 1 NSCLC with more frequent vascular and pleural invasions and a solid component more frequently found in ADCs. COPD promotes tumour proliferation and angiogenesis through the production of chemokines and cytokines, such as tumour necrosis factor-α, interleukin-1 and interleukin-6 [43]. Several studies have shown that the presence of a solid component is indicative of a more invasive profile [44,45]. One hypothesis is that when a tumour forms near an emphysematous or inflammatory lesion, the surrounding inflammation, including numerous cytokines (interleukin-6, tumour necrosis factor-α and interleukin-1b) [46] and chemokines (CXCL8 and CXCL1), alters the autocrine and paracrine interactions between malignant cells and invading leukocytes. Biton et al. showed that CD8 TIL exhaustion is correlated with COPD severity, whereas CD4 TIL levels remain stable [47]. This notion could explain the significant link between COPD status and ME. The CTV should be adjusted according to the COPD status.

**Figure 4:**
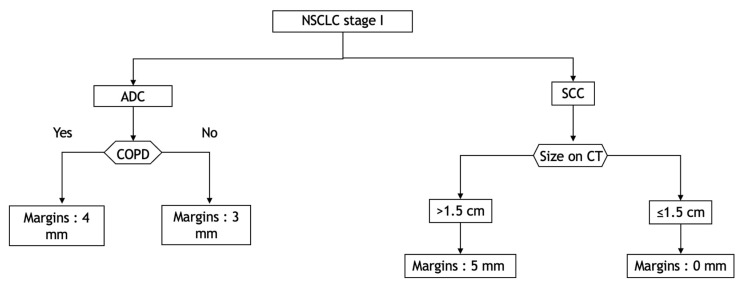
Decision tree according to histological type, COPD status and TIL rate to cover 95% of the distance of diffusion probability. ADC: adenocarcinoma; COPD: chronic obstructive pulmonary disease; CT: computed tomography; NSCLC: non-small-cell lung carcinoma; SCC: squamous cell carcinoma.

We believe that it might be prudent to apply a margin corresponding to a CTV according to the histological type, the COPD status for ADC, the rate of TILs and the tumour size for SCC. As an example, we propose a decision tree that could be a basis for deciding which margins to define. (Figure 4).

Our study is not exempt from bias, and the retraction of tissue during paraffin inclusion is a bias to consider. During the inclusion phase of surgical specimens, tissue shrinkage occurs. This retraction could lead to a decrease in the measured ME. Several other studies have shown tumour shrinkage at inclusion in NSCLC, head and neck cancers and breast cancers [15,48,49,50]. In a study of 100 head and neck cancer specimens, the average decreases in length, width and depth after formalin fixation were 4.40%, 6.18%, and 4.10%, respectively [48]. To overcome this uncertainty, we conducted a prospective study in 2017 that included 126 NSCLC surgical specimens that showed an average shrinkage of 4.06% after fixation [15]. We then applied this shrinkage coefficient to our measurements. The limited number of pathological slices per patient is also a limitation of our study; however, this finding is consistent with the literature [12,22]. Finally, the small number of patients in the subgroup analysis based on the TIL rate is also a limitation. Of note, we included only ADC and SCC, and the other histological types were not included based on the limited numbers. We were unable to study the relationship between ME and mutations, given the small number.

The mean value of ME was 2.18 mm for adenocarcinoma (ADC) and 1.33 mm for squamous cell carcinoma (SCC) (*p* = 0.001). However, considering 95% of the ME, margins of 7 mm and 5 mm must be allowed for ADC and SCC, respectively. The contribution of these CTV margins should be balanced against the good local controls in the literature [8,9,51,52].

## 5. Conclusions

The mean microscopic extension for all the samples examined was much lower than the CTV adopted by some previous studies and did not differ between the two histologic types studied.

In the case of stereotactic radiotherapy, in the light of our results, we believe that it might be prudent to apply a margin corresponding to a CTV according to the histological type, the COPD status for adenocarcinomas, the rate of TILs and the tumour size for squamous cell carcinomas. Larger prospective trials that take into consideration the tumour movement related to respiration by performing quadridimensional scanning and maximal intensity projection sequences should be performed.

## Figures and Tables

**Table 1:** Patient characteristics.

	Adenocarcinoma		Squamous Cell Carcinoma		Total	
	n	%	n	%	n	%
Patients	112	72	42	28	154	
Slides	341	73	127	27	468	
Age (mean, in years)	65.5		65.8			
Gender						
Male	52	46	33	79	85	55
Female	60	54	9	21	69	45
pTNM 2017						
pT1mi N0	7	6	0	0	7	4
pT1a N0	7	6	8	19	15	10
pT1b N0	43	39	13	31	56	36
pT1c N0	28	25	7	17	35	22
pT2a N0	27	24	14	33	44	28
Architecture						
Acinar	60	54				
Lepidic	18	16				
Mucinous	12	11				
Solid	10	9				
Papillary	7	6				
Micropapillary	3	3				
No Other Specified	1	1				
Atelectasis						
Yes	13	12	9	21	22	15
No	98	88	33	79	131	85
Site						
Proximal	16	14	8	19	24	16
Peripheral	95	85	34	81	129	84
Margins						
Nodular	32	29	16	38	48	31
Spiculated	79	71	26	62	105	62
Insufflation quality						
Good	55	49	24	57		
Medium	47	42	17	40		
Poor	10	9	1	3		
Angioinvasion						
Yes	10	8.9	7	17		
No	101	90	34	81		
Lymphatic invasion						
Yes	2	2	1	2		
No	109	97	41	98		
Fibrosis						
Yes	34	30	13	31		
No	78	70	29	69		
Haemorrhage						
Yes	44	39	17	40		
No	68	61	25	60		
Inflammation						
Yes	16	14	11	26		
No	96	86	31	74		
Mode of extension						
AIL	2	2	1	2	3	2
AIV	1	1	1	2	2	1
STAS	24	21	10	24	34	22
Interstitial	31	28	4	10	35	22
Tabacco						
Yes	87	78	39	93	126	82
No	24	21	3	7	27	18
COPD						
Yes	34	30	19	45	53	46
No	78	70	23	55	101	64
TILs						
0–10%	79	71	10	24	89	59
20–40%	27	24	25	60	52	33
50–90%	6	5	7	17	13	8

AIL: adjacent lymphatic invasion; AIV: adjacent vascular invasion; COPD: chronic obstructive pulmonary disease; STAS: spread through air space; TILs: tumour-infiltrating lymphocytes.

**Table 2:** Tumour size and microscopic extension according to histology.

	Mean (Standard Deviation)	Median (Q25–75)	Min	Max	n
ADC size (cm)	1.97 (0.848)	1.87 (1.35; 2.52)	0.21	3.95	112
ADC radiologic size (cm)	2.15 (0.909)	2.00 (1.50; 2.60)	0.80	5.30	112
SCC size (cm)	2.46 (1.00)	2.19 (1.50; 3.41)	1.04	4.16	42
SCC radiologic size (cm)	2.33 (1.10)	2.20 (1.57; 2.80)	0.30	5.40	42
ADC ME (mm)	0.734 (1.12)	0 (0; 1.30)	0	6.12	112
SCC ME (mm)	0.737 (1.49)	0 (0; 0.615)	0	5.94	42

ADC: adenocarcinoma; ME: microscopic extension; SCC: squamous cell carcinoma.

**Table 3:** Results of multivariate analysis to determine the statistical relationship between maximum distance ME and tumour size with the shrinkage coefficient for adenocarcinoma.

		Coefficients	*p*	*p* Global
Tumour size (mm)		0.0209 (−0.00802; 0.0595)	0.16	0.16
COPD	1 vs. 0	0.434 (−0.0260; 1.00)	0.065	0.065
Number of slides		0.166 (−0.0575; 0.504)	0.15	0.15
TILs (%)	20–40 vs. 0–10	0.0757 (−0.418; 0.517)	0.74	0.69
	50–90 vs. 0–10	−0.340 (−0.965; 0.377)	0.45	-
GTV volume (cm^3^)		−0.0304 (−0.0813; 0.0101)	0.21	0.21

COPD: chronic obstructive pulmonary disease; GTV: gross target volume; TILs: tumour-infiltrating lymphocytes.

**Table 4:** Results of multivariate analysis to determine the statistical relationship between maximum distance ME and tumour size with the shrinkage coefficient for squamous cell carcinoma.

		Coefficients	*p*	*p* Global
Tumour size (mm)		−0.0461 (−0.0681; −0.0170)	<0.01	<0.01
TILs (%)	0–10 vs. 20–40	0.979 (0.159; 2.41)	0.015	0.015
	50–90 vs. 20–40	−0.524 (−1.04; −0.172)	0.25	-
COPD	1 vs. 0	0.125 (−0.584; 1.08)	0.79	
Number of slides		−0.264 (−0.976; 0.287)	0.3	
GTV volume (cm3)		0.109 (0.0647; 0.169)	<0.001	

COPD: chronic obstructive pulmonary disease; GTV: gross target volume; TILs: tumour-infiltrating lymphocytes.

## Data Availability

Data available on request due to restrictions privacy. The data presented in this study are available on request from the corresponding author.

## Abbreviations

ADC	adenocarcinoma
BED	Biological Equivalent Dose
COPD	Chronic Obstructive Pulmonary Disease
CT	Computed Tomographic
CTV	Clinical Target Volume
DIAMIC	computerized database of anatomoclinical reports
ECE	Extra Capsular Extension
ESTRO	European Society of Radiation Oncology
GTV	Gross Tumour Volume
ICRU	International Commission on Radiation Units and Measurements
ITV	internal target volume
ME	microscopic tumour extension
NSCLC	Non-Small-Cell Lung Cancer
PTV	Planning Target Volume
SBRT	Stereotactic Body Radiation Therapy
SCC	Squamous Cell Carcinoma
TILs	Tumour-infiltrating Lymphocytes
WHO	World Health Organization

## References

[1] Herbst R.S., Heymach J.V., Lippman S.M. (2008). Lung Cancer. N. Engl. J. Med..

[2] Girard N., Mornex F. (2011). Stereotactic radiotherapy for non-small cell lung cancer: From concept to clinical reality. 2011 update. Cancer Radiother..

[3] Scott W.J., Howington J., Feigenberg S., Movsas B., Pisters K. (2007). American College of Chest Physicians Treatment of Non-Small Cell Lung Cancer Stage I and Stage II: ACCP Evidence-Based Clinical Practice Guidelines (2nd Edition). Chest.

[4] Chang J.Y., Mehran R.J., Feng L., Verma V., Liao Z., Welsh J.W., Lin S.H., O’Reilly M.S., Jeter M.D., Balter P.A. (2021). Stereotactic Ablative Radiotherapy for Operable Stage I Non-Small-Cell Lung Cancer (Revised STARS): Long-Term Results of a Single-Arm, Prospective Trial with Prespecified Comparison to Surgery. Lancet Oncol..

[5] Chang J.Y., Senan S., Paul M.A., Mehran R.J., Louie A.V., Balter P., Groen H.J.M., McRae S.E., Widder J., Feng L. (2015). Stereotactic Ablative Radiotherapy versus Lobectomy for Operable Stage I Non-Small-Cell Lung Cancer: A Pooled Analysis of Two Randomised Trials. Lancet Oncol..

[6] Zheng X., Schipper M., Kidwell K., Lin J., Reddy R., Ren Y., Chang A., Lv F., Orringer M., Kong S.F.-M. (2014). Survival Outcome after Stereotactic Body Radiation Therapy and Surgery for Stage I Non-Small Cell Lung Cancer: A Meta-Analysis. Int. J. Radiat. Oncol. Biol. Phys..

[7] Giraud P., Lacornerie T., Mornex F. (2016). Radiotherapy for primary lung carcinoma. Cancer Radiother..

[8] Fakiris A.J., McGarry R.C., Yiannoutsos C.T., Papiez L., Williams M., Henderson M.A., Timmerman R. (2009). Stereotactic Body Radiation Therapy for Early-Stage Non-Small-Cell Lung Carcinoma: Four-Year Results of a Prospective Phase II Study. Int. J. Radiat. Oncol. Biol. Phys..

[9] Ricardi U., Filippi A.R., Guarneri A., Giglioli F.R., Ciammella P., Franco P., Mantovani C., Borasio P., Scagliotti G.V., Ragona R. (2010). Stereotactic Body Radiation Therapy for Early Stage Non-Small Cell Lung Cancer: Results of a Prospective Trial. Lung Cancer.

[10] Vansteenkiste J., Crinò L., Dooms C., Douillard J.Y., Faivre-Finn C., Lim E., Rocco G., Senan S., Van Schil P., Veronesi G. (2014). 2nd ESMO Consensus Conference on Lung Cancer: Early-Stage Non-Small-Cell Lung Cancer Consensus on Diagnosis, Treatment and Follow-Up. Ann. Oncol..

[11] Timmerman R., Paulus R., Galvin J., Michalski J., Straube W., Bradley J., Fakiris A., Bezjak A., Videtic G., Johnstone D. (2010). Stereotactic Body Radiation Therapy for Inoperable Early Stage Lung Cancer. JAMA.

[12] Giraud P., Antoine M., Larrouy A., Milleron B., Callard P., De Rycke Y., Carette M.F., Rosenwald J.C., Cosset J.M., Housset M. (2000). Evaluation of Microscopic Tumor Extension in Non-Small-Cell Lung Cancer for Three-Dimensional Conformal Radiotherapy Planning. Int. J. Radiat. Oncol. Biol. Phys..

[13] Antoni D., Srour I., Mornex F. (2015). Lung cancer: Stereotactic body radiation therapy and surgery. Cancer Radiother..

[14] Waissi W., Noël G., Giraud P. (2015). Follow-up after lung stereotactic radiotherapy. Cancer Radiother..

[15] Park H.S., Lee S., Haam S., Lee G.D. (2017). Effect of Formalin Fixation and Tumour Size in Small-Sized Non-Small-Cell Lung Cancer: A Prospective, Single-Centre Study. Histopathology.

[16] Travis W.D., Brambilla E., Nicholson A.G., Yatabe Y., Austin J.H.M., Beasley M.B., Chirieac L.R., Dacic S., Duhig E., Flieder D.B. (2015). The 2015 World Health Organization Classification of Lung Tumors. J. Thorac. Oncol..

[17] Hendry S., Salgado R., Gevaert T., Russell P.A., John T., Thapa B., Christie M., van de Vijver K., Estrada M.V., Gonzalez-Ericsson P.I. (2017). Assessing Tumor Infiltrating Lymphocytes in Solid Tumors: A Practical Review for Pathologists and Proposal for a Standardized Method from the International Immuno-Oncology Biomarkers Working Group. Adv. Anat. Pathol..

[18] Li W., Jm Y., Gh L., Wx Z., Ww L., Bj Z. (2003). A Comparative Study on Radiology and Pathology Target Volume in Non-Small-Cell Lung Cancer. Zhonghua Zhong Liu Za Zhi.

[19] Chan R., He Y., Haque A., Zwischenberger J. (2001). Computed Tomographic-Pathologic Correlation of Gross Tumor Volume and Clinical Target Volume in Non-Small Cell Lung Cancer: A Pilot Experience. Arch. Pathol. Lab. Med..

[20] Yuan S., Meng X., Yu J., Mu D., Chao K.S.C., Zhang J., Zhong W., Yu Y., Wang J., Sun X. (2007). Determining Optimal Clinical Target Volume Margins on the Basis of Microscopic Extracapsular Extension of Metastatic Nodes in Patients with Non-Small-Cell Lung Cancer. Int. J. Radiat. Oncol. Biol. Phys..

[21] Loon J.V., Blauwgeers H., Rossi M., Klomp H., Gilhuijs K. (2012). Microscopic Disease Extension in Three Dimensions for Non-Small-Cell Lung Cancer: Development of a Prediction Model Using Pathology-Validated Positron Emission Tomography and Computed Tomography Features. Int. J. Radiat. Oncol. Biol. Phys..

[22] Grills I.S., Fitch D.L., Goldstein N.S., Yan D., Chmielewski G.W., Welsh R.J., Kestin L.L. (2007). Clinicopathologic Analysis of Microscopic Extension in Lung Adenocarcinoma: Defining Clinical Target Volume for Radiotherapy. Int. J. Radiat. Oncol. Biol. Phys..

[23] Guckenberger M., Andratschke N., Dieckmann K., Hoogeman M.S., Hoyer M., Hurkmans C., Tanadini-Lang S., Lartigau E., Romero A.M., Senan S. (2017). ESTRO ACROP Consensus Guideline on Implementation and Practice of Stereotactic Body Radiotherapy for Peripherally Located Early Stage Non-Small Cell Lung Cancer. Radiother. Oncol..

[24] Senthi S., Haasbeek C.J.A., Slotman B.J., Senan S. (2013). Outcomes of Stereotactic Ablative Radiotherapy for Central Lung Tumours: A Systematic Review. Radiother. Oncol..

[25] Trémolières P., Gonzalez-Moya A., Paumier A., Mege M., Blanchecotte J., Theotime C., Autret D., Dufreneix S. (2022). Lung Stereotactic Body Radiation Therapy: Personalized PTV Margins According to Tumor Location and Number of Four-Dimensional CT Scans. Radiat. Oncol..

[26] Chi A., Liao Z., Nguyen N.P., Xu J., Stea B., Komaki R. (2010). Systemic Review of the Patterns of Failure Following Stereotactic Body Radiation Therapy in Early-Stage Non-Small-Cell Lung Cancer: Clinical Implications. Radiother. Oncol..

[27] Wulf J., Baier K., Mueller G., Flentje M.P. (2005). Dose-Response in Stereotactic Irradiation of Lung Tumors. Radiother. Oncol..

[28] Klement R.J., Sonke J.-J., Allgäuer M., Andratschke N., Appold S., Belderbos J., Belka C., Blanck O., Dieckmann K., Eich H.T. (2020). Correlating Dose Variables with Local Tumor Control in Stereotactic Body Radiation Therapy for Early-Stage Non-Small Cell Lung Cancer: A Modeling Study on 1500 Individual Treatments. Int. J. Radiat. Oncol. Biol. Phys..

[29] Wilke L., Andratschke N., Blanck O., Brunner T.B., Combs S.E., Grosu A.-L., Moustakis C., Schmitt D., Baus W.W., Guckenberger M. (2019). ICRU Report 91 on Prescribing, Recording, and Reporting of Stereotactic Treatments with Small Photon Beams: Statement from the DEGRO/DGMP Working Group Stereotactic Radiotherapy and Radiosurgery. Strahlenther. Onkol..

[30] Pagès F., Berger A., Camus M., Sanchez-Cabo F., Costes A., Molidor R., Mlecnik B., Kirilovsky A., Nilsson M., Damotte D. (2005). Effector Memory T Cells, Early Metastasis, and Survival in Colorectal Cancer. N. Engl. J. Med..

[31] Angell H., Galon J. (2013). From the Immune Contexture to the Immunoscore: The Role of Prognostic and Predictive Immune Markers in Cancer. Curr. Opin. Immunol..

[32] Fridman W.H., Pagès F., Sautès-Fridman C., Galon J. (2012). The Immune Contexture in Human Tumours: Impact on Clinical Outcome. Nat. Rev. Cancer.

[33] Galon J., Costes A., Sanchez-Cabo F., Kirilovsky A., Mlecnik B., Lagorce-Pagès C., Tosolini M., Camus M., Berger A., Wind P. (2006). Type, Density, and Location of Immune Cells within Human Colorectal Tumors Predict Clinical Outcome. Science.

[34] Donnem T., Hald S.M., Paulsen E.-E., Richardsen E., Al-Saad S., Kilvaer T.K., Brustugun O.T., Helland A., Lund-Iversen M., Poehl M. (2015). Stromal CD8+ T-Cell Density—A Promising Supplement to TNM Staging in Non-Small Cell Lung Cancer. Clin. Cancer Res..

[35] Geng Y., Shao Y., He W., Hu W., Xu Y., Chen J., Wu C., Jiang J. (2015). Prognostic Role of Tumor-Infiltrating Lymphocytes in Lung Cancer: A Meta-Analysis. CPB.

[36] Buisseret L., Desmedt C., Garaud S., Fornili M., Wang X., Van den Eyden G., de Wind A., Duquenne S., Boisson A., Naveaux C. (2017). Reliability of Tumor-Infiltrating Lymphocyte and Tertiary Lymphoid Structure Assessment in Human Breast Cancer. Mod. Pathol..

[37] Mani N.L., Schalper K.A., Hatzis C., Saglam O., Tavassoli F., Butler M., Chagpar A.B., Pusztai L., Rimm D.L. (2016). Quantitative Assessment of the Spatial Heterogeneity of Tumor-Infiltrating Lymphocytes in Breast Cancer. Breast Cancer Res..

[38] Bohan P.M.K., Chick R.C., Hickerson A.T., Messersmith L.M., Williams G.M., Cindass J.L., Lombardo J., Collins R., Brady R.O., Hale D.F. (2020). Correlation of Tumor Microenvironment from Biopsy and Resection Specimens in Untreated Colorectal Cancer Patients: A Surprising Lack of Agreement. Cancer Immunol. Immunother..

[39] Nakahara Y., Mochiduki Y., Miyamoto Y., Nakahara Y., Katsura Y. (2005). Prognostic Significance of the Lymphocyte-to-Neutrophil Ratio in Percutaneous Fine-Needle Aspiration Biopsy Specimens of Advanced Nonsmall Cell Lung Carcinoma. Cancer.

[40] Yoon H.J., Kang J., Park H., Sohn I., Lee S.-H., Lee H.Y. (2020). Deciphering the Tumor Microenvironment through Radiomics in Non-Small Cell Lung Cancer: Correlation with Immune Profiles. PLoS ONE.

[41] Sun R., Limkin E.J., Vakalopoulou M., Dercle L., Champiat S., Han S.R., Verlingue L., Brandao D., Lancia A., Ammari S. (2018). A Radiomics Approach to Assess Tumour-Infiltrating CD8 Cells and Response to Anti-PD-1 or Anti-PD-L1 Immunotherapy: An Imaging Biomarker, Retrospective Multicohort Study. Lancet Oncol..

[42] Tang C., Hobbs B., Amer A., Li X., Behrens C., Canales J.R., Cuentas E.P., Villalobos P., Fried D., Chang J.Y. (2018). Development of an Immune-Pathology Informed Radiomics Model for Non-Small Cell Lung Cancer. Sci. Rep..

[43] Maeda R., Tomita M., Usuda K., Uramoto H. (2019). Clinicopathologic Characteristics of Non-Small Cell Lung Cancer in Patients with Smoking-Related Chronic Obstructive Pulmonary Disease. Gen. Thorac. Cardiovasc. Surg..

[44] Riquet M., Foucault C., Berna P., Assouad J., Dujon A., Danel C. (2006). Prognostic Value of Histology in Resected Lung Cancer with Emphasis on the Relevance of the Adenocarcinoma Subtyping. Ann. Thorac. Surg..

[45] Barletta J.A., Yeap B.Y., Chirieac L.R. (2010). Prognostic Significance of Grading in Lung Adenocarcinoma. Cancer.

[46] Brusselle G.G., Joos G.F., Bracke K.R. (2011). New Insights into the Immunology of Chronic Obstructive Pulmonary Disease. Lancet.

[47] Biton J., Ouakrim H., Dechartres A., Alifano M., Mansuet-Lupo A., Si H., Halpin R., Creasy T., Bantsimba-Malanda C., Arrondeau J. (2018). Impaired Tumor-Infiltrating T Cells in Patients with Chronic Obstructive Pulmonary Disease Impact Lung Cancer Response to PD-1 Blockade. Am. J. Respir. Crit. Care Med..

[48] Chen C.-H., Hsu M.-Y., Jiang R.-S., Wu S.-H., Chen F.-J., Liu S.-A. (2012). Shrinkage of Head and Neck Cancer Specimens after Formalin Fixation. J. Chin. Med. Assoc..

[49] Horn C.L., Naugler C. (2014). Breast Specimen Shrinkage Following Formalin Fixation. Pathol. Lab. Med. Int..

[50] Hsu P.-K., Huang H.-C., Hsieh C.-C., Hsu H.-S., Wu Y.-C., Huang M.-H., Hsu W.-H. (2007). Effect of Formalin Fixation on Tumor Size Determination in Stage I Non-Small Cell Lung Cancer. Ann. Thorac. Surg..

[51] Timmerman R.D., Paulus R., Pass H.I., Gore E.M., Edelman M.J., Galvin J., Straube W.L., Nedzi L.A., McGarry R.C., Robinson C.G. (2018). Stereotactic Body Radiation Therapy for Operable Early-Stage Lung Cancer: Findings from the NRG Oncology RTOG 0618 Trial. JAMA Oncol..

[52] Videtic G.M., Paulus R., Singh A.K., Chang J.Y., Parker W., Olivier K.R., Timmerman R.D., Komaki R.R., Urbanic J.J., Stephans K.L. (2019). Long-Term Follow-up on NRG Oncology RTOG 0915 (NCCTG N0927): A Randomized Phase 2 Study Comparing 2 Stereotactic Body Radiation Therapy Schedules for Medically Inoperable Patients with Stage I Peripheral Non-Small Cell Lung Cancer. Int. J. Radiat. Oncol. Biol. Phys..

